# Accuracy Verification of Magnetic Resonance Imaging (MRI) Technology for Lower-Limb Prosthetic Research: Utilising Animal Soft Tissue Specimen and Common Socket Casting Materials

**DOI:** 10.1100/2012/156186

**Published:** 2012-04-24

**Authors:** Mohammad Reza Safari, Philip Rowe, Arjan Buis

**Affiliations:** ^1^Department of Orthotics and Prosthetics, University of Social Welfare and Rehabilitation Sciences, Tehran, Iran; ^2^National Centre for Prosthetics and Orthotics, University of Strathclyde, Glasgow G4 0LS, UK; ^3^Department of Bioengineering, University of Strathclyde, Glasgow G4 0NW, UK

## Abstract

Lower limb prosthetic socket shape and volume consistency can be quantified using MRI technology. Additionally, MRI images of the residual limb could be used as an input data for CAD-CAM technology and finite element studies. However, the accuracy of MRI when socket casting materials are used has to be defined. A number of six, 46 mm thick, cross-sections of an animal leg were used. Three specimens were wrapped with Plaster of Paris (POP) and the other three with commercially available silicone interface liner. Data was obtained by utilising MRI technology and then the segmented images compared to corresponding calliper measurement, photographic imaging, and water suspension techniques. The MRI measurement results were strongly correlated with actual diameter, surface area, and volume measurements. The results show that the selected scanning parameters and the semiautomatic segmentation method are adequate enough, considering the limit of clinical meaningful shape and volume fluctuation, for residual limb volume and the cross-sectional surface area measurements.

## 1. Introduction

The “quality” of the socket fit, providing the coupling between the skeleton of the residual limb and the rigid structure of the prosthesis, is vital to the overall success of the prosthetic replacement. This quality is the most important characteristic of lower limb prostheses as indicated by prosthetic users [1–3].

State of the art prosthetic sockets is designed and handcrafted individually. The socket is usually made through the process of shape capturing, rectification, and alignment. Depending on the socket concept, a Plaster of Paris (POP) wrap cast is manually applied over the residual limb (residuum) or over the elastomeric liner covering the residual limb with the aim to capture a modified shape of the soft tissues. This shape is used to produce a positive model, which is afterwards adapted (rectified) according to one of a number of design paradigms. These procedures are highly individual, often inconsistent, and based on tacit knowledge. The performance by an individual prosthetist will be strongly influenced by personal experience, skill, and beliefs [4, 5]. When the socket design process is not reproducible, it will, besides the obvious prosthetic fit issues, affect the positioning of the socket relative to the prosthetic foot (alignment) and hence alter ambulation. Without doubt, those difficulties compromise the prosthetic rehabilitation process [4–6].

The evaluation of the shape capturing process and subsequent rectification process in terms of repeatability of inter- and intrasocket volume and shape comparison is notoriously complicated, due to the difficulty in establishing an accurate and reliable reference grid. It was reported that it is feasible to use the tibia bone, the only rigid entity of the residuum, as a reference grid for 3D alignment of multiple MRI images [7] and spiral X-ray computed tomography (SXCT) scans [8–10]. Both SXCT and MRI technologies are capable of providing two-dimensional (2D) and three-dimensional (3D), soft, and hard (bone) tissue images of the residual limb.

Additionally, 3D images from both systems have been used in the production of prosthetic sockets using computer-aided design and manufacturing (CAD-CAM) technology and finite element (FE) studies [11–17]. The CAD offers a way to do what prosthetists have been doing manually in a computerised way and, hence, could be considered having consistency due to the nature of the approach. The digital shape data is saved into the computer, and the prosthetist uses the computer programme to rectify the shape for the final socket fit.

Optical scanning, including laser scanning, is the most commonly used method in residual limb scanning. However, errors may occur in the shape capturing process and/or socket manufacturing from the data. Reliability, repeatability, and accuracy of two common CAD shape techniques (contact method; Tracer CAD and noncontact method; T-ring) were evaluated using three models and compared to the conventional plaster shape capturing method [18]. Reliability of Tracer CAD and T-ring was high (ICC > 0.999 and ICC > 0.984 resp.). Both CAD systems showed an inaccuracy and lack of repeatability especially at the distal deformable part of the residual limb models. MRI can be utilised in CAD system as this provides information on both internal and external structure of the residual limb, particularly when the soft tissue stress-strain information, and hence the FE methods, can be incorporated into residual limb geometric data to get to the desirable socket fit.

MRI is a nonionising, high-resolution imaging technique which can provide a clear contrast between the various tissues within the residuum. Numerous studies have reported that MRI is an accurate measurement method and has been utilised to acquire morphological information of different tissues, for example, bone, muscle, and articular cartilage [19–28]. However, the accuracy of MRI soft tissue geometric measurement when enclosed within casting materials has not been investigated.

The accuracy of MRI measurement, besides to voxel size and scanning protocol, depends on the accuracy of segmentation process. The segmentation process can be performed manually, automatically, or semiautomatically. Although automated segmentation has less inter/intraobserver variation due to less operator intervention [29], several studies have shown that manual segmentation is a reliable method [27, 30–32]. In Semiautomatic segmentation, a seed point is placed within the area of interest, and then the threshold is set so that the boundary is detected automatically. If there was an error in borders, it is corrected using the edit tool.

Residual limb is normally wrapped with casting material in order to prevent soft tissue distortion due to gravitational forces during scanning. The close proximity of the casting material to the soft tissue could influence the residual limb boundary detection during segmentation process. The accuracy of residual limb skin-surrounding boundary detection in MRI images depends on the image signal intensity as well as on possible distortional effect (e.g., chemical shift artefact) resulting from fat and/or socket material. In the Patella tendon bearing casting concept, just the POP is used, whereas, in pressure cast, the limb is surrounded by silicone, and then POP covers the silicone liner. The signal intensity of silicone and POP doped with 1 gr/lit copper sulphate (CU) was measured in MRI images in previous study [33]. The silicone has 1.5 times stronger signal intensity in MRI than fresh POP + 1 gr/lit CU. Therefore, there is a possibility that each material influences the skin boundary detection differently and, hence, affects the soft tissue morphologic measurements. This would be exaggerated when the subcutaneous adipose tissue superimposes, due to chemical shift artefact, to the surrounding casting material.

A prerequisite for using MRI technology in the quantification of the volume and shape of the residual limb is to determine whether commonly used prosthetic materials have an artefact on the scanned data. Buis et al. reported on a study where commonly used socket materials were examined using MRI technology. The results show that POP spiked with 1 gr/lit CU and elastomeric liner material (silicone) did not show a significant chemical shift artefact [7].

The aim of the current study was to use animal specimens to verify the accuracy of MRI, using Semiautomatic segmentation process, in relation to cross-sectional dimension, surface area, and volume measurement when specimens are enclosed within POP and a commercially available silicone interface liner.

## 2. Methods

**Figure 1:**
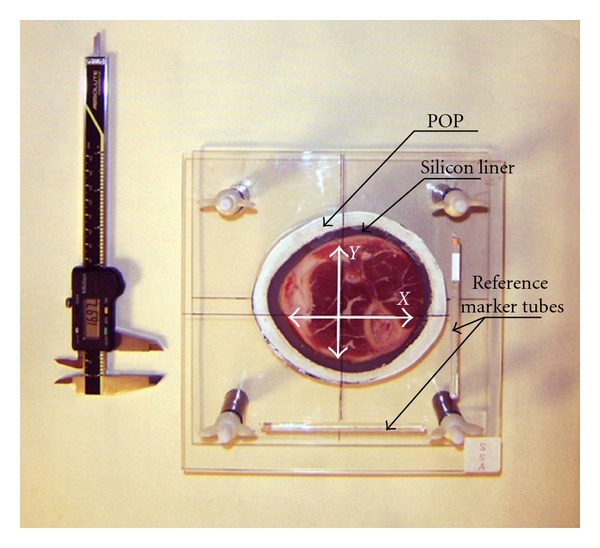
Animal specimen within Perspex fixator rig wrapped with silicon liner and POP. The *X* and *Y* cross-sectional diameters were defined at the midpoint of the reference marker tubes.

Number of Six, 46 mm thick, different diameter, and fresh cross-sections of an animal (hind leg of a pork) at transtibial level, were individually sandwiched between two optically clear 10 mm thick Perspex plates using a column drill machine with a depth travel limiter, to guarantee parallel positioning of the plates with a constant spacing of 46 mm. This allowed the application of a silicone liner material ring and POP + 1 gr/lit CU around the specimen. After curing of the POP, the plates were secured parallel to each other by means of four plastic bolts and wing nuts placed at each corner of the perspex plates. Three specimens were wrapped with POP only and three with silicon liner rings and POP. All specimens were prepared a day before scanning and were kept moisturised and isolated from air exposure to prevent the POP from drying out (Figure 1).

Afterwards, each side of the specimen was photographed, using a digital camera and then scanned using the MRI machine. Then, cross-sectional diameters of each specimen were measured using a digital vernier calliper and compared to the both MRI and photographic derived values. After that the photographic images of cross-sectional surface area of the specimens were measured using Adobe Photoshop CS3 Extended software and then compared with the measurement in the corresponding MRI images. In addition, the volume of each specimen was compared using the water suspension method and MRI volume measurement. Precise details and reasons for these procedures now follow.

Magnetic susceptibility difference between adjacent substances inside the magnetic field results in microscopic gradients or variation in the magnetic field strength near or across the interface of two materials. This artefact, known as a “susceptibility artefact,” will result in bright and dark areas with spatial shifting of surrounding material. To eliminate the susceptibility artifact, it was suggested to use a material which has the same magnetic susceptibility as water. Perspex is a substance which can be used for this purpose [34].

**Figure 2:**
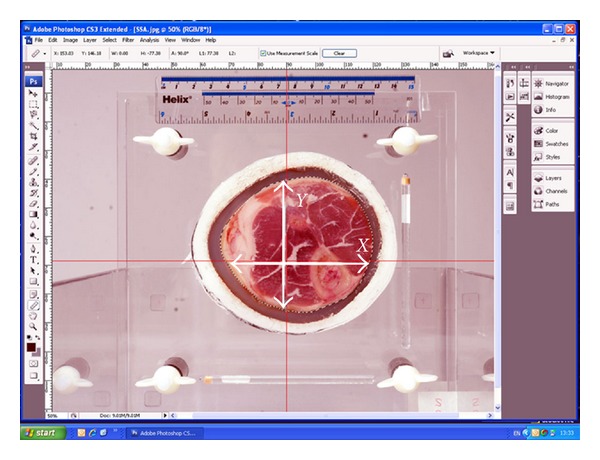
Diameter and surface area measurement of specimen using Adobe Photoshop Extended version.

**Figure 3:**
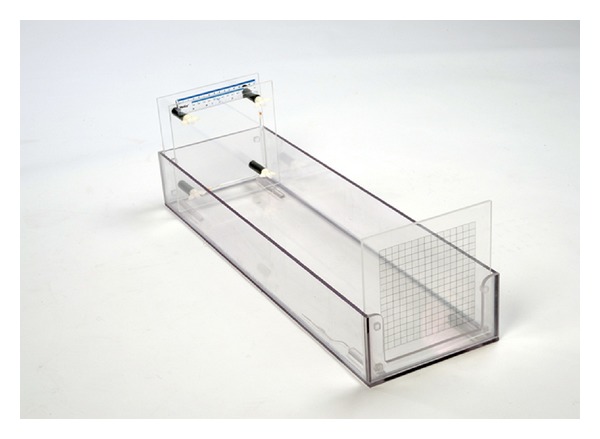
Grid used for adjusting of angle and distance of camera from specimens and consistent photography of all specimens.

For each specimen, one of the perspex plates contained two marker tubes (100 mm L × 5 mm Ø) positioned perpendicular to each other for reference purposes in the MRI images. The marker tubes were filled with water doped with 1 gr/lit CU to enhance contrast. Additionally, a measurement ruler was attached to the surface of the perspex plate for scaling purposes of the photographic images (Figure 2). The cross-sections of all specimens were photographed with a digital camera so that the surface area of cross-sectional specimen could be measured and compared with that of MRI measurements. To avoid any photographic distortion of the actual specimen, the axis of the camera lens was positioned in line with the centre of the specimen with the aid of a purpose designed rig (Figure 3). The rig consisted out of a 600 mm long and 200 mm wide perspex box that would allow placement of a sandwiched specimen to be inserted at the long end of the box on one site, and a digital camera on the other. This setup enabled a consistent distance between camera and the object of interest.

 The smallest achievable voxel size using the 3 Tesla MRI scanner is 1.17 × 1.17 × 0.6 mm with slice thickness of 1.2 mm by scanning 300 mm length of the limb. The sagital fast spoiled gradient-recalled-echo (FSPGR) pulse sequence with the following parameters was adopted: field intensity 3T, repetition time 6.9 s, time of echo 1.5 s, inversion time 500 ms, bandwidth 31.25 KHz, flip angle 12 deg, matrix 256 × 256, slice thickness 1.2 mm, voxel dimensions 1.17 × 1.17 × 0.6 mm, and a 1 signal average.

In order to examine the accuracy of measurements derived from photographic images, cross-sectional diameters of each specimen were measured from the origin of the *X* and *Y* directions of each surface of the perspex plates, black cross hairs in Figure 1, using a digital vernier calliper.

Photographic images were downloaded to Adobe Photoshop CS3 Extended software and scaled according the ruler placed on the Perspex fixation plates (Figure 2). The cross-sectional dimensions of the specimen were measured in the *X* and *Y* direction from the origin of the Perspex plates, red crosshairs in (Figure 2). This was followed by tracing the specimen boundary (skin) with the “magnetic lasso” tool of the software. This lasso tool is especially useful for the automatic selection of objects with complex edges set against objects with different contrasts. Edges were refined further to adjust for errors in boundary selection. Subsequently, the total of the surface area of the specimen was given by the software.

After MRI imaging, volume of each specimen was measured using the water suspension technique based on Archimedes' principle. This involved suspension of an object below the surface of the water in a container placed on an electronic scale with an accuracy of 1 gram. To a first approximation, the volume of the immersed object is simply the increase in weight divided by the density of the fluid [35]. Animal specimens were not frozen during this procedure. Specimens were immersed in water for a maximum of one minute to be able to read the scale. This is a relatively short period of time; hence, the possibility of water absorption by specimen was minimal and could be neglected [36, 37].

**Figure 4:**
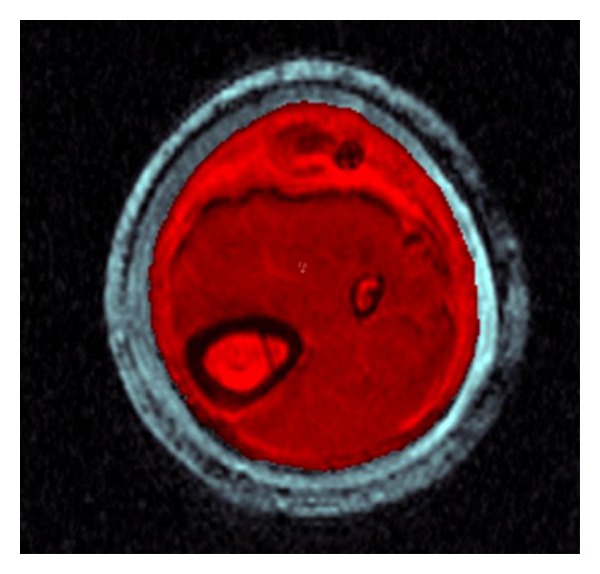
Semiautomatic segmentation of the surface area of test specimen.

**Figure 5:**
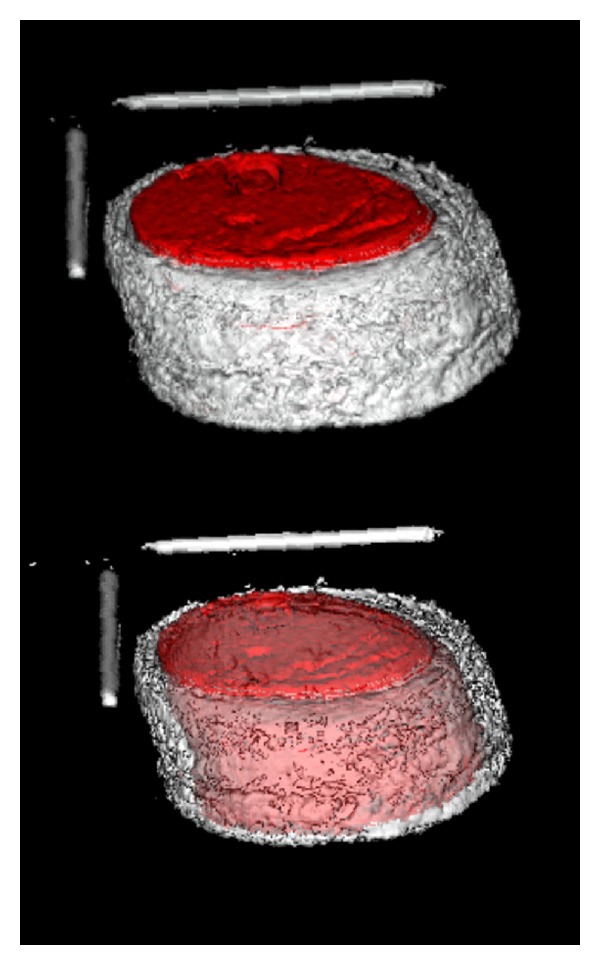
Volume render object map of test specimen.

Recorded MRI data was transferred to the Analyze software for comparative analysis with photographic and real specimen data. There are three options available within the software to segment the object of interest, namely, manual, Semiautomatic, and automatic, respectively. Automatic segmentation involves morphology erosion and dilation with the attempt to automatically segment an object within a volume. Since the signal intensity of the subcutaneous fat and the silicone liner are close to each other, automatic segmentation was not appropriate because the software is not able to differentiate between the two. Therefore, a Semiautomatic segmentation process was selected. A so called “seeding” point was placed within the area of interest in the first slice of the volume, and a threshold value was set so that the boundary was detected automatically (Figure 4). This procedure was repeated for each slice of the whole volume and saved as an object map, (Figure 5).

**Figure 6:**
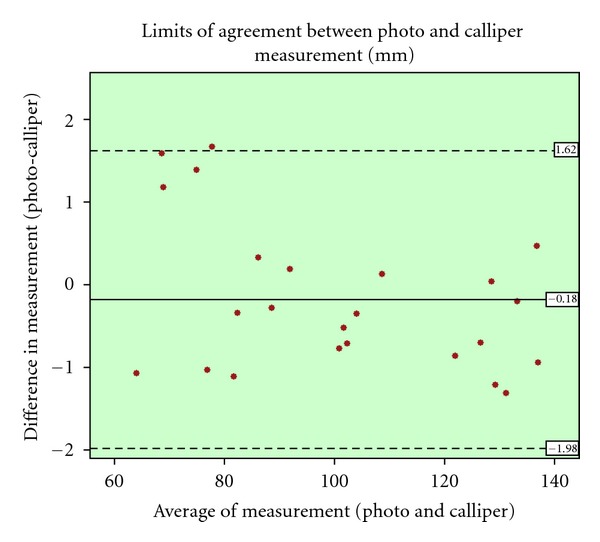
Bland and Altman plot for the diameter measurement comparison of vernier calliper and photo.

 Cross-sectional diameter of the specimen was measured using the calliper software tool. Additionally, the surface area of the cross-sections of specimens in contact with the perspex plates and volume results of the scanned specimens were calculated using the corresponding object maps. Then the MRI diameter measurements were compared with the calliper measurements, and the cross-sectional surface area data were compared to that of the photographic image results. The MRI volume measurements were also compared to that of water suspension method. The photographic dimensional measurement, as a virtual environment, was compared to actual calliper measurement. The three image sources, MRI, photographic and vernier calliper were registered in the same reference grid, using two glass tube reference markers, before measurements were recorded.

paired *t*-test and pearson correlation coefficient were used for the statistical analysis of the results. Additionally the Bland and Altman plot was utilised [38]. This is method for assessing conformity, between two methods of measurement, by plotting the difference of those methods against the average of the two. The mean difference is set as a dashed horizontal line, and the limits of agreement are set from that line, plus/minus 1.96 times the standard deviation (mean ± 1.96 sd) as illustrated in Figure 6 to Figure 9.

## 3. Results

The minimum, maximum, and mean difference and absolute difference, pearson correlation coefficient, the results of paired samples *t*-test significance and 95% confidence Interval of the difference are summarised in Table 1. The photographic diameter measurement is correlated strongly with calliper diameter measurement (*r* = 0.99, *P* = 0.00), and there was not a statically significant difference between two measurements using paired samples *t*-test (95% CI: −0.56 to 0.19). The difference in measurements was plotted against the average of two measurements. A higher density is observed below the mean line in the Bland and Altman plot (Figure 6).

**Figure 7:**
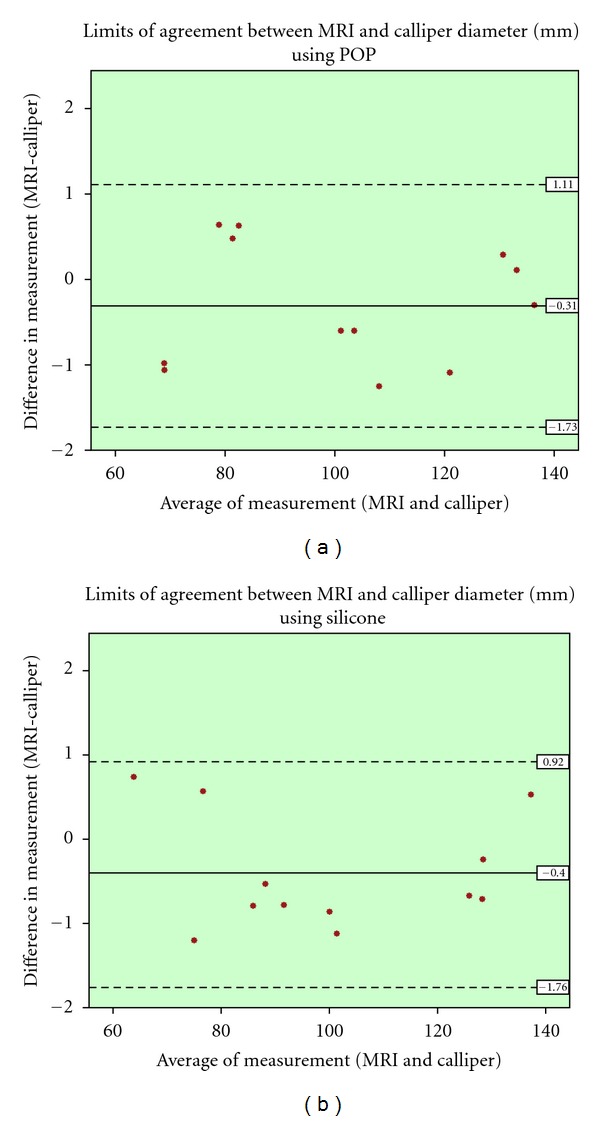
Bland and Altman plot for the diameter measurement comparison of vernier calliper and MRI; (a): POP and (b): silicone.

There was a strong correlation between MRI and calliper measurement methods when specimens were wrapped with either POP (*r* = 1.000, *P* = 0.00) or silicone (*r* = 1.000, *P* = 0.00), and the results of paired samples *t*-test showed no statically significant different between two methods (POP: 95% CI, −0.14 to 0.76 and silicone: 95% CI, −0.01 to 0.84). In the Bland and Altman plots, the data was spread randomly within the limits of agreement for POP wrapped specimens, while higher density of difference value was concentrated below the mean line for the silicone wrapped specimens (Figure 7).

**Figure 8:**
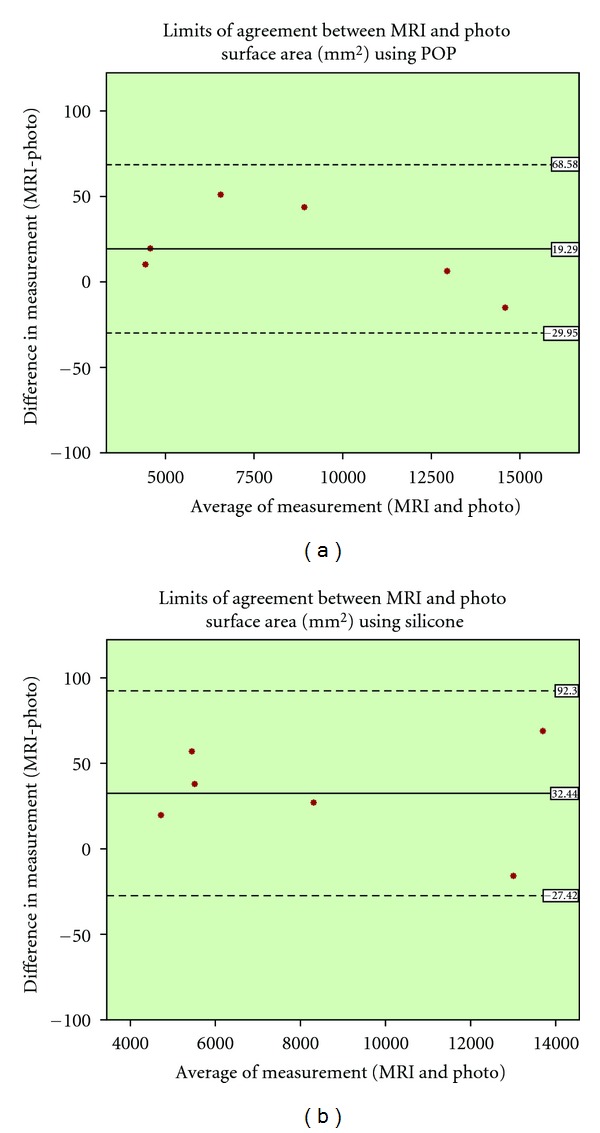
Bland and Altman plot for the surface area measurement comparison of MRI and photo, (a): POP and (b): silicone.

 The surface area measurement of MRI and photographic measurements were strongly correlated for both POP and silicone interventions (*r* = 1.00, *P* = 0.00). Additionally, there was no statically significant difference between means while the POP was used (95% CI, 8.70 to 43.05). However, MRI and photographic measurement were significantly different when silicone was used (95% CI, 1.03 to 63.85). Besides, the Bland and Altman plot show that absolute difference increases with increasing surface area in silicone wrapped specimens (Figure 8).

**Figure 9:**
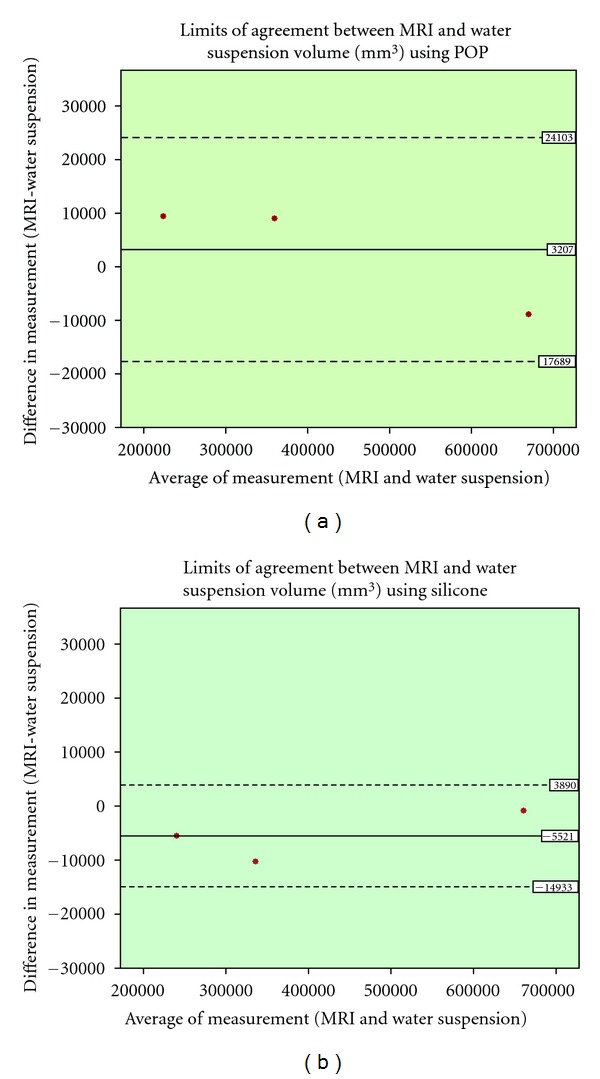
Bland and Altman plot for the volume measurement comparison of MRI and water suspension, (a): POP and (b): silicone.

The MRI volume measurement was correlated strongly with water suspension volume measurement when both casting materials were utilised (*r* = 1.00, *P* = 0.00). Additionally, there was not a statically significant difference between MRI and water suspension methods (POP: 95% CI, −17689 to 24103 and silicone: 95% CI, −14933 to 3890). Differences of measurements were plotted against the average of two measurements. No noticeable trend could be observed in the Bland and Altman plot (Figure 9).

## 4. Discussion

This study explored the potential of MRI technology for prosthetic socket research. The accuracy of this technology is dependant, besides chemical and mechanical shift issues on voxel size, image resolution, and segmentation procedure. Higher resolution and smaller voxel size require an increase in the duration of a single scan. Previous pilot studies indicated that the maximum scanning time that is considered reasonable for a volunteer subject should not be longer than 10 minutes. The compromise between scanning duration and an accurate/sensitive enough voxel size and resolution in this study resulted in an eight and a half minutes scanning sequence. The semiautomatic segmentation procedure, employed in this study, permits fine adjustment for errors occurring, in parts of the image with close signal intensity, during the seeding procedure. During scanning, the residual limb is normally wrapped with casting material to prevent soft tissue distortion due to gravitational forces. The accuracy of residual limb boundary detection, besides to the image artefact, depends on the signal intensity of surrounding materials. In this study, the accuracy of MRI in soft tissue dimension, surface area, and volume measurements, when soft tissue is enclosed with common casting materials, was examined. This has not been investigated previously.

Results show that MRI is an accurate method for measuring soft tissue cross-sectional diameter, surface area, and volume, Table 1. These results are in line with a study published by Beneke et al. [28]. The study reports on a similar project where the cross-sections of a cadaveric thigh muscle was measured at two points, lower and upper thigh, using MRI and photographic images. Results highlighted differences of 1.2% which are slightly elevated compared to this study. This can be attributed to a limited number of images as well as poor control of the variation of the angle of the MRI and photographical plane. In addition, freezing of the cadaver, after the MRI scan, may change the cross-sectional dimensions of the soft tissue that was photographed [28]. Engstrom et al., [19] measured serial cross-sections of cadaveric thigh muscles and reported that MRI measurement provided accurate and precise estimation of surface area of most thigh muscles. Similar to Beneke's study, this higher error is attributed to the freezing of the cadaver after MRI and before photography. Additional errors might be related to the limited number of images used and difficulties experienced of identifying the boundary of closely opposed muscles in the images that were segmented separately, whereas, in the present study the whole soft tissue boundary was traced resulting in a smaller error.

Validation of MRI volume measurement is normally performed by using phantoms, comprised of materials other than soft tissue and bone, to be able to compare the actual volume of phantoms with that of MRI images. It is reported by Mitsiopoulos et al. [25] that the volume of phantoms measured by MRI indicated an error less than 1%. However, there is a note of caution, the material used for the tested phantoms is not a representation for soft tissue. Cyteval et al. [21] measured vertebral body dimensions using MRI, in order to compare the MRI measurement of vertebral area and volume with direct cadaver measurements. In their study, water displacement was used for volume measurement of cadaver vertebra. The intraclass correlation coefficient between MRI and suspension methods was 0.95. It was concluded that MRI is a feasible, reproducible, and accurate method for area and volume measurement of vertebral bodies. Furthermore, Mitsiopoulos and his colleagues [25] showed that MRI can provide an accurate area and volume measurement of the skeletal muscle and adipose tissue-free skeletal muscle (ATFSM). The ATFSM area in 119 images (38.9 ± 22.3) and cadaver (39.5 ± 23.0) were not different. It was shown that the MRI volume estimates are in good agreement with those of cadaver sections [25] (correlation for regression analysis was 0.98 to 0.99 for all variables, *P* < 0.001).

In order to identify the proper time for the permanent prosthetic fitting, Lilja and Oberg measured post-amputation volume fluctuation of the residual limb using laser scanning. Based on amputees experience, they assumed the “bad fit” criteria to be one or two layers of socks required over the residual limb, that is, if using one or two socks by the amputee was required, then a new socket must be made. They measured the percentage volume of the one and two socks over the residual limb as to be 5.2% and 9.4% [39]. Their results of sock volume percentage are in agreement with that of Fernie and Holliday [40]. The mean volume difference between MRI and actual measurement when the specimens were wrapped with POP and silicone were 1.85% POP and −1.79%, respectively. This suggests that the adopted MRI settings and image processing procedure, that is, voxel size and segmentation process, are accurate enough to locate the clinical meaningful volume difference between two volume images of the residual limb. This also suggests that MRI could provide accurate enough input data to be used in CAD systems as well as FE methods.

Additionally, Sanders et al. calculated the uniform volume change of 5% in a limb with 90 mm diameter that would be 1 mm change in diameter [41]. The mean difference between MRI and actual diameter measurements was −0.31 mm and −0.42 mm for POP and silicone wrapped specimens, respectively, hence, small enough to find clinical significant diameter difference between two volume images.

There was not a statistically significant difference between any MRI and actual value except for MRI surface area and the photographic measurement in silicone wrapped specimens despite the small mean difference percentage. The results indicate that the silicone wrapped specimens, compared to the POP wrapped ones, resulted in more consistent MRI diameter, surface area, and volume measurements as the standard deviations of measurements are smaller relative to the mean difference values; however, the POP wrapped specimens had larger mean differences compared to silicone wrapped specimens. This could be explained by the signal intensity difference of two materials as the silicone showed 1.5 times stronger signal intensity in MRI than fresh POP doped with 1 gr/lit CU [33]. Furthermore, the close vicinity of material with the skin made the boundary detection difficult, particularly with the existence of subcutaneous adipose tissue chemical shift which, in some parts, superimposed to the surrounding material. This could be avoided in MRI images of the residual limb if a small gap could be made between skin and material using some noncompressible, MRI-opaque materials for example, prosthetic socks.

The total number of twelve diameters, six cross-sectional surface areas, and three volume measurements were performed in this study. The sample size was relatively small, However, considering the strong correlation between MRI and actual measurements, this sample size may be satisfying [42, 43]. However, the power calculation using the results of this study reveals that larger sample size is required to achieve higher statistical power for the *t*-test.

## 5. Conclusion

The cross-sectional diameter, surface area, and total volume of the evaluated animal specimen were accurately measured using MRI technology. The results show that the selected scanning parameters and the Semiautomatic segmentation method are adequate enough, considering the limit of clinical meaningful shape and volume fluctuation, for residual limb volume and the cross-sectional surface area measurements while wrapped with silicon liner and/or POP.

The correct residual limb geometry is essential for the accuracy of FE studies as well as CAD-CAM technology; hence, errors in segmentation of the residual limb from surrounding POP, when MRI image is used as an input data, will jeopardize results. The results of this study indicate that the scanning parameter besides the segmentation process is accurate enough for FE studies and CAD-CAM technology.

One should note that the number of scanned specimen is limited in this study due to time and funding constraints. Moreover, the automatic segmentation was not employed in this study because of the close proximity of the signal intensity of casting material and underlying skin. Therefore, examining the inter- and intrarater reliability of Semiautomatic segmentation procedure is suggested for the future studies.

## Figures and Tables

**Table 1:** The minimum, maximum, and mean difference and absolute difference, Pearson Correlation Coefficient, the results of paired samples *t*-test significance, and 95% confidence interval of the difference.

Measurement difference	Material	*n*	Absolute difference				Difference				Correlation coefficient (*P*)*	*P* (95% CI of the difference)
			Min (%)	Max (%)	Mean (%)	SD	Min (%)	Max (%)	Mean (%)	SD		
Calliper—photo diameter measurement (mm)	NA	24	0.04 (0.03)	1.67 (2.29)	0.77 (0.85)	0.49	−1.31 (−1.29)	1.67 (1.65)	−0.18 (−0.17)	0.09	0.999 (0.000)	0.326 (−0.56, 0.19)

MRI—photo diameter measurement (mm)	POP	12	0.11 (0.08)	1.25 (1.53)	0.66 (0.73)	0.36	1.25 (−1.53)	0.61 (0.81)	−0.31 (−0.33)	0.71	1.000 (0.000)	0.16 (−0.14, 0.76)
	Silicone	12	0.24 (0.19)	1.20 (1.59)	0.73 (0.79)	0.26	−1.20 (−1.59)	0.74 (1.17)	−0.42 (−0.41)	0.67	1.000 (0.000)	0.053 (−0.01, 0.84)

MRI—photo Surface area measurement (mm^2^)	POP	6	6.33 (0.05)	51.03 (0.78)	24.31 (0.35)	18.54	−15.06 (−0.10)	51.03 (0.78)	19.29 (0.31)	24.62	1.000 (0.000)	0.113 (−6.55, 45.13)
	Silicone	6	15.83 (0.12)	68.87 (1.04)	37.72 (0.51)	21.25	−15.83 (−0.12)	68.87 (1.04)	32.44 (0.47)	29.93	1.000 (0.000)	0.045 (1.03, 63.85)

MRI—water suspension volume measurement (mm^3^)	POP	3	8855 (1.31)	9443 (4.31)	9110 (2.72)	301	−8855 (−1.31)	9443 (4.31)	3207 (1.85)	10448	1.000 (0.000)	0.648 (−17689, 24103)
	Silicone	3	831 (0.13)	10243 (3.00)	5521 (1.79)	4706	−10243 (−3.00)	−831 (−0.13)	−5521 (−1.79)	4706	1.000 (0.009)	0.179 (−14933, 3890)

*Correlation is significant at alpha = 0.01.

## References

[1] Legro MW, Reiber G, del Aguila MD (1999). Issues of importance reported by persons with lower limb amputations and prostheses. *Journal of Rehabilitation Research and Development*.

[2] Hoaglund FT, Jergesen HE, Wilson L (1983). Evaluation of problems and needs of veteran lower-limb amputees in the San Francisco Bay area during the period 1977–1980. *Journal of Rehabilitation Research and Development*.

[3] Hagberg K, Brånemark R, Hägg O (2004). Questionnaire for Persons with a Transfemoral Amputation (Q-TFA): initial validity and reliability of a new outcome measure. *Journal of Rehabilitation Research and Development*.

[4] Convery PP, Buis AWP, Wilkie R, Sockalingam S, Blair A, McHugh B (2003). Measurement of the consistency of patellar-tendon-bearing cast rectification. *Prosthetics and Orthotics International*.

[5] Buis AWP, Blair A, Convery P, Sockalingam S, McHugh B (2003). Pilot study: data-capturing consistency of two trans-tibial casting concepts, using a manikin stump model: a comparison between the hands-on PTB and hands-off ICECAST Compact concepts. *Prosthetics and Orthotics International*.

[6] Johansson S, Öberg T (1998). Accuracy and precision of volumetric determinations using two commercial CAD systems for prosthetics: a technical note. *Journal of Rehabilitation Research and Development*.

[7] Buis AWP, Condon B, Brennan D, McHugh B, Hadley D (2006). Magnetic resonance imaging technology in transtibial socket research: a pilot study. *Journal of Rehabilitation Research and Development*.

[8] Commean PK, Smith KE, Cheverud JM, Vannier MW (1996). Precision of surface measurements for below-knee residua. *Archives of Physical Medicine and Rehabilitation*.

[9] Jilin L, Ruwen Z, Susu R, Weikang G (1997). Calibration and analysis of measurement system for surface of the stump. *Chinese Journal of Biomedical Engineering*.

[10] Smith KE, Commean PK, Vannier MW (1996). Residual-limb shape change: three-dimensional CT scan measurement and depiction in vivo. *Radiology*.

[11] Faulkner VW, Walsh NE (1989). Computer designed prosthetic socket from analysis of computed tomography data. *Japan Patent Office*.

[12] Zachariah SG, Sanders JE, Turkiyyah GM (1996). Automated hexahedral mesh generation from biomedical image data: applications in limb prosthetics. *IEEE Transactions on Rehabilitation Engineering*.

[13] Commean PK, Smith KE, Vannier MW, Szabo BA, Actis RL (1997). Finite element modeling and experimental verification of lower extremity shape change under load. *Journal of Biomechanics*.

[14] Shuxian Z, Wanhua Z, Bingheng L (2005). 3D reconstruction of the structure of a residual limb for customising the design of a prosthetic socket. *Medical Engineering and Physics*.

[15] Zhang M, Mak AFT, Roberts VC (1998). Finite element modelling of a residual lower-limb in a prosthetic socket: a survey of the development in the first decade. *Medical Engineering and Physics*.

[16] Lee WCC, Zhang M, Jia X, Cheung JTM (2004). Finite element modeling of the contact interface between trans-tibial residual limb and prosthetic socket. *Medical Engineering and Physics*.

[17] Udai AD, Sinha AN Processing magnetic resonance images for CAD model development of prosthetic limbs socket.

[18] McGarry A (2009). *Evaluation of the Tracer Cad and T Ring Prosthetic Shape Capture Systems*.

[19] Engstrom CM, Loeb GE, Reid JG, Forrest WJ, Avruch LA (1991). Morphometry of the human thigh muscles. A comparison between anatomical sections and computer tomographic and magnetic resonance images. *Journal of Anatomy*.

[20] Aisen AM, Martel W, Braunstein EM (1986). MRI and CT evaluation of primary bone and soft tissue tumors. *American Journal of Roentgenology*.

[21] Cyteval C, Thomas E, Picot MC, Derieffy P, Blotman F, Taourel P (2002). Normal vertebral body dimensions: a new measurement method using MRI. *Osteoporosis International*.

[22] Eckstein F, Sittek H, Milz S, Putz R, Reiser M (1994). The morphology of articular cartilage assessed by magnetic resonance imaging (MRI). Reproducibility and anatomical correlation. *Surgical and Radiologic Anatomy*.

[23] Haubner M, Eckstein F, Schnier M (1997). A non-invasive technique for 3-dimensional assessment of articular cartilage thickness based on MRI part 2: validation using CT arthrography. *Magnetic Resonance Imaging*.

[24] McGibbon CA (2003). Inter-rater and intra-rater reliability of subchondral bone and cartilage thickness measurement from MRI. *Magnetic Resonance Imaging*.

[25] Mitsiopoulos N, Baumgartner RN, Heymsfield SB, Lyons W, Gallagher D, Ross R (1998). Cadaver validation of skeletal muscle measurement by magnetic resonance imaging and computerized tomography. *Journal of Applied Physiology*.

[26] Mortimore IL, Marshall I, Wraith PK, Sellar RJ, Douglas NJ (1998). Neck and total body fat deposition in nonobese and obese patients with sleep apnea compared with that in control subjects. *American Journal of Respiratory and Critical Care Medicine*.

[27] Walton JM (1997). Measurement of the quadriceps femoris muscle using magnetic resonance and ultrasound imaging. *British Journal of Sports Medicine*.

[28] Beneke R, Neuerburg J, Bohndorf K (1991). Muscle cross-section measurement by magnetic resonance imaging. *European Journal of Applied Physiology and Occupational Physiology*.

[29] Douglas TS, Solomonidis SE, Lee VSP, Spence WD, Sandham WA, Hadley DM (1999). Automatic segmentation of magnetic resonance images of the trans- femoral residual limb. *Medical Engineering and Physics*.

[30] Gomberg BR, Saha PK, Wehrli FW (2005). Method for cortical bone structural analysis from magnetic resonance images. *Academic Radiology*.

[31] Tracy BL, Ivey FM, Metter EJ, Fleg JL, Siegel EL, Hurley BF (2003). A more efficient magnetic resonance imaging-based strategy for measuring quadriceps muscle volume. *Medicine and Science in Sports and Exercise*.

[32] Zentai CP, Worku D, Tuncali K Validation of 3D assessment of MR imaging-guided precutaneous crytherapy of a soft-tissue metastasis.

[33] Safari MR (2010). *Inter- and Intra Socket Shape and Volume Consistency Assessed Using Magnetic Resonance Imaging for Hands-on and Hands-off Casting of Amputee below Knee Sockets*.

[34] Moerland MA, Beersma R, Bhagwandien R, Wijrdeman HK, Bakker CJG (1995). Analysis and correction of geometric distortions in 1.5 T magnetic resonance images for use in radiotherapy treatment planning. *Physics in Medicine and Biology*.

[35] Hughes SW (2005). Archimedes revisited: a faster, better, cheaper method of accurately measuring the volume of small objects. *Physics Education*.

[36] Cunningham HM, Lawrence GA (1977). Effect of exposure of meat and poultry to chlorinated water on the retention of chlorinated compounds and water. *Journal of Food Science*.

[37] Kent M, Knöchel R, Daschner F, Berger UK (2001). Composition of foods including added water using microwave dielectric spectra. *Food Control*.

[38] Bland JM, Altman DG (1986). Statistical methods for assessing agreement between two methods of clinical measurement. *The Lancet*.

[39] Lilja M, Oberg T (1997). International forum: proper time for permanent prosthetic fitting. *Japan Patent Office*.

[40] Fernie GR, Holliday PJ (1982). Volume fluctuations in the residual limbs of lower limb amputees. *Archives of Physical Medicine and Rehabilitation*.

[41] Sanders JE, Mitchell SB, Zachariah SG, Wu K (2003). A digitizer with exceptional accuracy for use in prosthetics research: a technical note. *Journal of Rehabilitation Research and Development*.

[42] Algina J, Olejnik S (2003). Sample size tables for correlation analysis with applications in partial correlation and multiple regression analysis. *Multivariate Behavioral Research*.

[43] Cohen J (1988). *Statistical Power Analysis for the Behavioral Sciences*.

